# Genetically predicted dietary macronutrient intakes and atrial fibrillation risk: a Mendelian randomization study

**DOI:** 10.1186/s40001-024-01781-z

**Published:** 2024-04-12

**Authors:** Zhuoya Zhang, Jiale Zhang, Haoyang Jiao, Wei Tian, Xu Zhai

**Affiliations:** 1 Affiliated Hospital of Nanjing University of Chinese Medicine, Nanjing, China; 2 Institute of Basic Theory for Chinese Medicine, China Academy of Chinese Medical Sciences, Beijing, 100700 China; 3 Institute of Documentation, China Academy of Chinese Medical Sciences, Beijing, China; 4 Gaoyang County Hospital, Baoding, 071599 Hebei Province China; 5 Guanganmen Hospital, China Academy of Chinese Medical Sciences, Beijing, China

**Keywords:** Cardiovascular disease, Dietary macronutrient intake, Atrial fibrillation risk, Mendelian randomization

## Abstract

**Background and aim:**

Previous observational investigations have indicated a potential association between relative dietary macronutrient intakes and atrial fibrillation and flutter (AF) risk. In this study, we employed Mendelian Randomization (MR) to evaluate the presence of causality and to elucidate the specific causal relationship.

**Methods:**

We employed six, five, and three single nucleotide polymorphisms (SNPs) as instrumental variables for relative carbohydrate, protein, and fat intake, identified from a genome-wide association study that included 268,922 individuals of European descent. Furthermore, we acquired summary statistics for genome-wide association studies on AF from the FinnGen consortium, which involved 22,068 cases and 116,926 controls. To evaluate the causal estimates, we utilized the random effect inverse variance weighted method (IVW) and several other MR methods, including MR-Egger, weighted median, and MR-PRESSO, to confirm the robustness of our findings.

**Results:**

Our analysis indicates a convincing causal relationship between genetically predicted relative carbohydrate and protein intake and reduced AF risk. Inverse variance weighted analysis results for carbohydrates (OR = 0.29; 95% CI (0.14, 0.59); *P* < 0.001) and protein (OR = 0.47; 95% CI (0.26, 0.85); *P* = 0.01) support this association. Our MR analysis did not identify a significant causal relationship between relative fat intake and AF risk.

**Conclusion:**

Our study provides evidence supporting a causal relationship between higher relative protein and carbohydrate intake and a lower risk of atrial fibrillation (AF).

## Introduction

Atrial fibrillation (AF) is a common arrhythmia typically caused by electrical abnormalities within the heart, leading to irregular contractions of the upper chambers (atria) and impaired pumping function [1]. AF affects approximately 33 million people globally and is the most common cardiac arrhythmia. According to estimates, by 2050, 6–12 million people worldwide will be affected by this disease, and by 2060, Europe will have 17.9 million people suffering from this condition [2]. AF is a significant risk factor for stroke, with AF patients having more than a fivefold increased risk compared to non-AF patients [3]. Its etiology may be related to hypertension, coronary artery disease, diabetes, thyroid disorders, pulmonary disease, and certain cardiac conditions [4]. Lifestyle factors such as excessive alcohol intake, smoking, physical inactivity, and obesity have also been implicated in AF development [5]. Although the pathogenesis of AF is partly understood, a definitive cure for AF has not been identified, making prevention and risk factor control crucial.

Dietary macronutrients refer to three major nutrients: carbohydrates, proteins, and fats [6, 7]. The proportions and amounts of macronutrient intakes can influence health and the risk of diseases. A balanced diet with appropriate intakes of macronutrients is key to maintaining health and reducing disease risk [8]. Many studies have shown a close association between macronutrient intakes and cardiovascular risk. A traditional Mediterranean diet emphasizing high-quality carbohydrates and proteins and a controlled intake of high fats benefits cardiovascular protection [9, 10]. Although higher carbohydrate intake seems associated with increased cardiovascular risks in some Asian populations, the evidence is inconsistent [11]. While low-carbohydrate diets effectively induce short-term weight loss, their long-term safety and effects remain unclear [12]. Recent studies report conflicting results regarding how carbohydrate intake influences atrial fibrillation (AF) risk [13]. For example, some show that reducing carbohydrate intake may increase AF risk by 16–18%, whereas others indicate that high carbohydrate intake at breakfast could decrease cardiovascular disease risk [14]. Regular consumption of low-carbohydrate and high-protein diets without accounting for carbohydrate quality or protein sources is associated with elevated cardiovascular risk [15]. Furthermore, some studies have found that low saturated fat intake can lower the incidence of atrial fibrillation and other cardiovascular diseases [16, 17]. However, other studies have presented opposing results [18, 19]. Above all, numerous observational studies have explored the association between macronutrient intakes and the risk of AF, but their results are not entirely consistent. Due to the inability of observational studies to control for confounding factors, it is difficult to establish a causal relationship between macronutrient intakes and the risk of atrial fibrillation. Therefore, it is essential to employ Mendelian Randomization (MR) to investigate the genuine causal relationship between macronutrient intakes and the risk of AF.

**Fig. 1:**
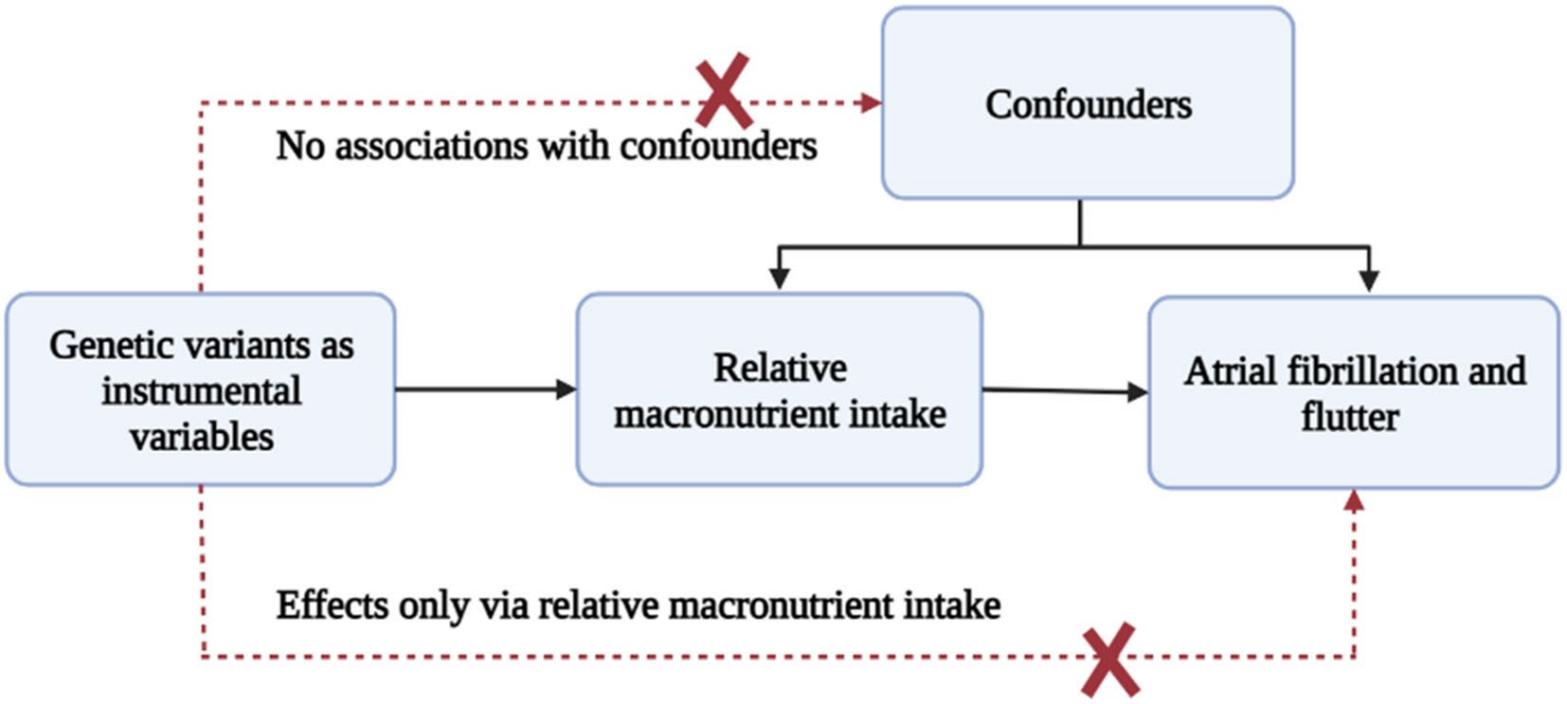
Mendelian randomization analyses to investigate associations between relative macronutrient intakes and the risk of atrial fibrillation and flutter. The broken lines signify potential causal effects (pleiotropic or direct) between variables that would violate the assumptions of Mendelian randomization

Mendelian Randomization (MR) is an epidemiological methodology that utilizes genetic variants as instrumental variables to circumvent confounding factors and reverse causality. [20] The three core assumptions of MR are instrumental variable assumption, independence assumption and exclusion restriction assumption. Similar to a randomized trial, genotypes are randomized at conception, making MR immune to confounding and reverse causality as per Mendel's second law [21]. Consequently, MR is a robust tool for predicting causal associations, as illustrated in Fig. 1. This study employs MR analysis to explore the relationship between macronutrient intakes and AF by utilizing genetically predicted relative macronutrient intakes as the exposure variable.

## Materials and methods

### Study design

Our study utilized a two-sample MR approach to examine the causal association between the risk of atrial fibrillation and flutter and the proportion of macronutrients consumed. This study adhered to the guidelines for the Preferred Reporting of Observational Studies in Epidemiology Using Mendelian Randomization (The STROBE-MR Statement) [22]. Our investigations relied on publicly accessible GWAS summary data, which had already undergone ethical review board approvals before their inclusion in our study.

### Instruments selection for relative macronutrients intake

We obtained GWAS summary statistics from a large genome-wide association study that included 268,922 individuals of European ancestries aged 27–71 years for relative intake of macronutrients [23], the most recent GWAS on relative macronutrients intake based on the European populations when we started the MR analysis. In the original GWAS study, relative macronutrients intake was expressed as % of total energy intake (E%) and assessed using comprehensive food-item questionnaires including previous-day or habitual dietary intake.

As genetic instruments, we selected nucleotide polymorphisms (SNPs) that demonstrated a strong association with relative macronutrients intake at a genome-wide significance level (p < 5 × 10^−8^). We, respectively, used 6 SNPs strongly associated with relative carbohydrate intake, 5 SNPs with relative protein intake, and 3 SNPs with relative fat intake as instrumental variables for the corresponding exposures. To maintain independence, we performed a linkage disequilibrium (LD) test (r^2^ < 0.001 and distance > 10 000 kb) [24]. We did not use palindromic SNPs with intermediate allele frequencies that pair with each other since they may invert the direction of a causal effect. We utilized proxy SNPs as substitutes for the corresponding SNPs only if they were not present in the outcome data. The proxy SNPs were identified by searching the SNIPA database (https://snipa.helmholtz-muenchen.de/snipa3/index.php). We performed a manual inspection using PhenoScanner for screening association with confounder [25]to exclude confounders. In screening for confounders, we focused on factors known to influence AF pathogenesis, including anatomical deformities (e.g., left atrium dilation and hypertrophic cardiopathy), valvular heart diseases, hyperthyroidism, chronic inflammation, infections, familial predispositions, and the use of specific medications such as statins and ACEIs/ARBs. This meticulous process helped refine our SNP selection, aiming for a clearer understanding of macronutrient intake's impact on AF risk. The R^2^ was then utilized to evaluate the extent to which each SNP accounted for the variance in relative macronutrient intake. [26]. To evaluate the effectiveness of instrumental variables, we utilized the F statistic, considering an F value greater than 10 as indicative of strong instruments [27].

### Genetic associations with atrial fibrillation and flutter

Our GWAS summary statistics for AF were obtained from the FinnGen project, which can be accessed at https://gwas.mrcieu.ac.uk/datasets/finn-b-I9_AF. There were 22,068 cases of atrial fibrillation and flutter and 116,926 people who served as controls in this study.

### Statistical analysis

We conducted all MR analyses using R software (version 4.2.2), with the Mendelian Randomization [28], RadialMR [29], and MR-PRESSO [30] packages utilized for the MR analyses. The primary MR analyses were conducted using inverse variance weighted (IVW) analyses [31]. We used the Cochran Q test to assess the heterogeneity of IVW estimates, with P < 0.05 indicating significant heterogeneity. In addition, we conducted several sensitivity analyses to assess the robustness of the results. Specifically, we employed five different MR methods, namely MR-Egger [32], weighted median [33], and MR-PRESSO [30], which have been widely used in MR studies. The MR-Egger method, a popular approach for detecting and correcting pleiotropy, was utilized to estimate the potential impact of pleiotropy on the result. *P* < 0.05 from the MR-Egger intercept indicates the presence of directional pleiotropy, which may bias the MR estimates [32]. The weighted median approach was used to generate a more reliable estimate, even when up to 50% of the instruments were invalid, by taking the median of all possible pairwise IVW estimates [33]. To enhance the reliability and validity of our results, we employed the MR-PRESSO analysis, which detects and corrects for outliers and horizontal pleiotropy, was used to assess the potential impact of outliers on the results [30]. For each MR method, we estimated the causal effect of AF as odds ratios (OR) per one standard deviation (SD) increase in genetically predicted continuous traits, along with corresponding 95% confidence intervals (CI). A significance threshold of *P* < 0.05 was used to determine statistical significance. Additionally, we conducted leave-one-out analyses to assess the impact of each SNP on the overall causal estimates. This approach involves systematically removing one SNP at a time and re-analyzing the data to evaluate the sensitivity of the results to each SNP.

## Results

**Fig. 2:**
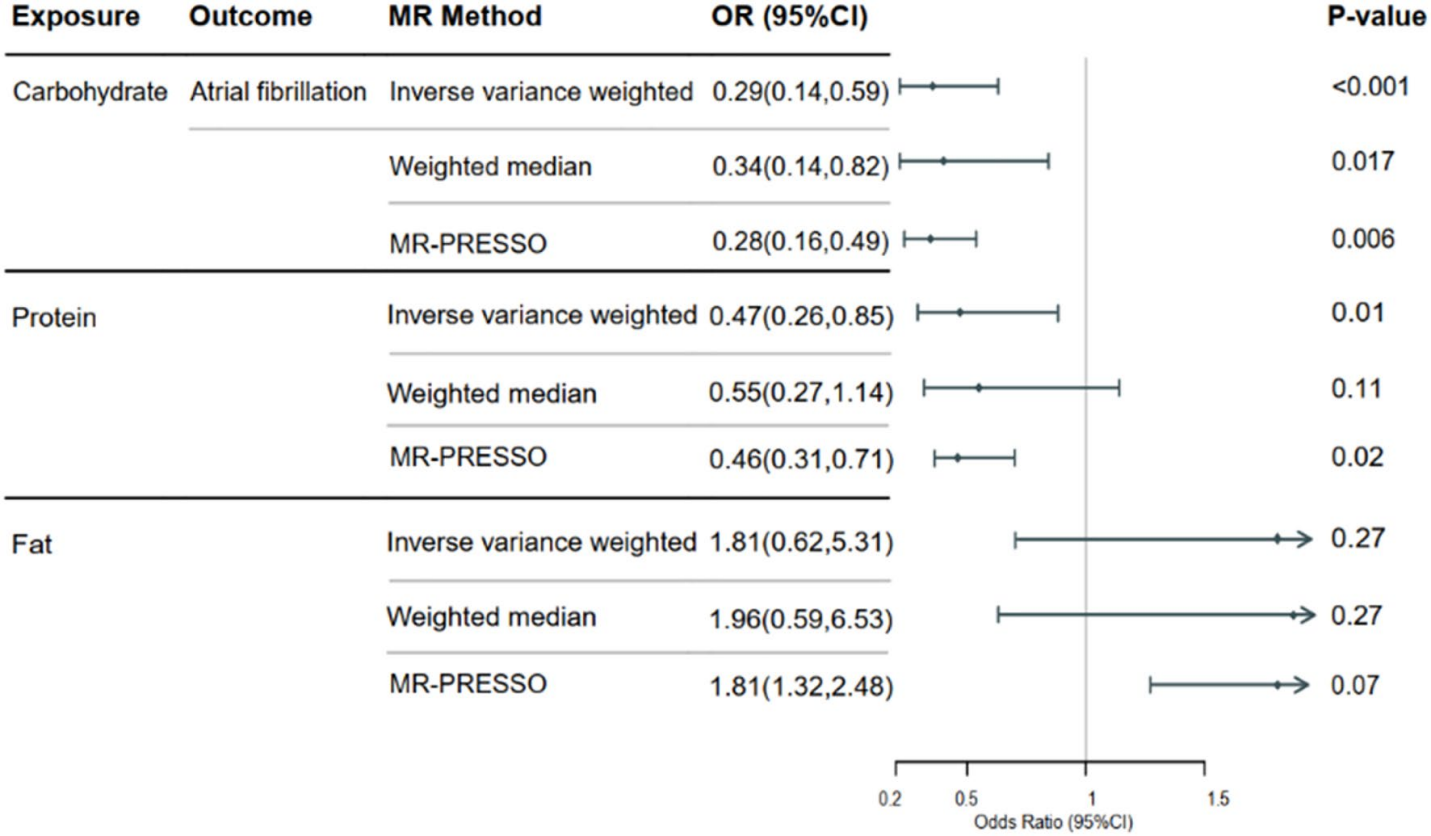
The odds ratios (OR) depicting the associations between genetically predicted relative macronutrient intake and AF risk are presented. *CI* confidence interval; *MR* Mendelian randomization

**Fig. 3:**
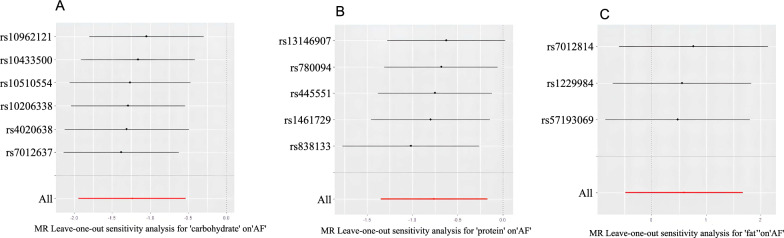
The leave-one-out analyses revealed no significant differences in the estimated causal effects when individual SNPs were removed from our MR analysis. **A** MR leave-one-out sensitivity analysis for ' carbohydrate ' on 'AF'. **B** MR leave-one-out sensitivity analysis for 'protein' on 'AF'. **C** MR leave-one-out sensitivity analysis for ' fat ' on 'AF'

Our analysis utilizing the inverse variance weighted (IVW) method revealed a robust causal association between genetically predicted relative carbohydrate and protein intake and a decreased risk of AF as illustrated in Fig. 2. For all three macronutrients, the F-values of the instrumental variables exceeded 10, indicating a robust association with relative macronutrient intake. Our results were unlikely confounded by heterogeneity or pleiotropic effects, as demonstrated by the absence of significant heterogeneity of inverse variance weighted (IVW) estimates (*Q*-test, *P* > 0.05) and directional pleiotropy (MR-Egger intercept, *P* > 0.05). Based on our leave-one-out analyses (Fig. 3), we found no significant differences in the estimated causal effects when individual SNPs were excluded, underscoring the robustness of our MR analysis. Consequently, the estimated effect cannot be ascribed to any particular SNP. Our study suggests that genetically predicted higher relative carbohydrate intake and protein intake may protect against the risk of AF. Our analysis did not identify a significant causal relationship between relative fat intake and AF risk.

## Discussion

We conducted a Mendelian Randomization (MR) analysis using a sample of 22,068 cases of atrial fibrillation and flutter and 116,926 controls to investigate the potential causal relationship between genetically predicted relative carbohydrate and protein intake and atrial fibrillation (AF), while minimizing confounding and reverse causation often observed in observational studies. Acknowledging the inherent limitations of our approach, it's important to note that despite employing various MR methods to mitigate the influence of pleiotropy, the potential for unmeasured confounding variables and pleiotropic effects persists. To account for potential pleiotropy, we employed various MR methods, each with different assumptions of pleiotropy, and the consistent results from these methods provided robust support for causal inference. Our analysis revealed a significant inverse association between relative carbohydrate and protein intake and AF risk. In contrast, our MR analysis did not identify a significant causal relationship between relative fat intake and AF risk. These findings suggest that increasing protein and carbohydrate intake from various nutrients may be a promising modifiable factor for preventing atrial fibrillation and highlight the importance of dietary modifications in preventing and managing AF. However, further studies are needed to fully elucidate the exact mechanisms underlying the protective effects of relative carbohydrate and protein intake on AF.

Multiple studies [34, 35] have demonstrated that lifestyle interventions can reduce AF incidence and mortality, particularly in high-risk populations. Recently, research has found that weight loss through enhanced management of risk factors can alleviate the symptom burden and severity of AF [36]. Therefore, lifestyle interventions [37] are crucial in reducing the risk of cardiovascular diseases, including AF. In addition to rhythm control, rate control, and anticoagulation therapy, changes in lifestyle and risk factors, including dietary structure and exercise, have been recognized as another pillar of AF treatment [38, 39].

Several studies have investigated the link between carbohydrates and AF. The latest research, published in J Am Heart Assoc [13], reveals that a carbohydrate-restricted diet may increase the risk of AF. Researchers evaluated the link between carbohydrate consumption and AF events in this large, community-based, prospective cohort study involving 13,384 participants. During a 22.4-year follow-up period, 1,808 AF cases (13.5%) were recorded. The study found that individuals on a low-carbohydrate diet may be associated with an increased risk of AF. Conversely, an increase in carbohydrate intake by 9.4% was associated with an 18% reduction in the incidence. Furthermore, we found some studies linking protein intake to cardiovascular disease. Higher plant protein consumption was associated with a modest reduction in overall and cardiovascular mortality risk [40]. Interestingly, a 2016 study [41] found that higher animal protein intake was positively associated with cardiovascular mortality. According to a study presented at the American College of Cardiology’s Annual Scientific Session and the World Congress of Cardiology,^1^ compared with women who consumed less protein, women who consumed moderately higher than the recommended daily amount of protein had a significantly lower likelihood of developing atrial fibrillation.^2^

Possible underlying mechanisms may explain that carbohydrate and protein intakes may be protective factors against AF risk. Irregular heartbeats characterize AF, and myocardial cells require more energy to maintain normal mechanical and electrical function. Mitochondria are essential organelles in cardiac cells responsible for converting nutrients into energy to meet the high energy demands of the heart. However, the increased metabolic rate of AF requires high energy metabolism, which may lead to mitochondrial overwork and oxidative stress, resulting in mitochondrial damage [42]. Recent research has indicated a close association between mitochondrial dysfunction and the development of AF [43]. Mitochondrial dysfunction may cause energy metabolism disorders in myocardial cells [44] and changes in calcium dynamics [45] and further exacerbate the development of AF. In addition, mitochondrial dysfunction may also lead to increased oxidative stress and inflammation reaction [46] and cell apoptosis in myocardial cells [47]. Studies have shown that improving myocardial mitochondrial function can alleviate the occurrence of AF [48]. Carbohydrates are the primary fuel for myocardial mitochondrial energy production. Carbohydrates are broken down into glucose in the body, then converted into adenosine triphosphate (ATP) through the glycolysis pathway, an important energy source supporting heart function. Amino acids are the basic units of proteins. They produce ATP in mitochondria by forming α-ketoglutarate or entering the tricarboxylic acid (TCA) cycle. In particular, the branched-chain amino acid leucine is an important energy source for the myocardium [49]. Therefore, appropriate carbohydrate and protein intakes may alleviate the progression of AF by improving myocardial mitochondrial function.

In addition, carbohydrate intake can also affect insulin levels, which is a hormone that regulates blood glucose. High insulin levels can promote glucose uptake and utilization, increasing the body's energy levels. Moderate carbohydrate intake can help maintain stable blood glucose levels, reducing the incidence of AF. However, excessive carbohydrate intake may lead to elevated blood glucose levels, negatively impacting cardiovascular health. Numerous studies have also confirmed the negative impact of carbohydrates on cardiovascular diseases [50]. High blood glucose levels can lead to cardiovascular diseases such as atherosclerosis, hypertension, and heart disease, which may also affect heart rate stability [51].

The source of carbohydrates and protein is also a noteworthy focus. High-quality carbohydrates refer to those that are abundant in nutrients such as dietary fiber, vitamins, and minerals [52]. These nutrients have the potential to decrease blood pressure, mitigate the risk of cardiovascular disease, and safeguard the cardiovascular system. Studies [53, 54] have shown that a moderate intake of whole grains, fruits, vegetables, and other carbohydrates can lower the risk of cardiovascular disease. Whole grain foods, such as oats, whole wheat bread, and brown rice, are healthy sources of carbohydrates. One important indicator is the low glycemic index (GI), meaning the digestion speed is slower and provides longer-lasting energy. Foods with a low GI can aid in managing and regulating blood glucose [55]. A study lasting from 12 to 26 weeks has shown that a low GI diet gradually enhances blood sugar control and insulin sensitivity, reducing the risk of heart attacks [56, 57]. Given that AF is a form of cardiovascular disease, it may benefit from adopting a low GI diet. Conversely, consuming ultra-processed foods (UPFs), including high-sugar foods, sweetened beverages, and poor-quality proteins, may harm cardiovascular health [58]. Excessive intake can cause a sharp rise and fall in blood sugar levels, increasing cardiovascular disease risk. UPFs usually contain many additives and high-calorie food ingredients, which may increase the release of inflammatory factors in the body with long-term consumption [59]. Furthermore, the source of protein emphasized the importance of high-quality protein. Red meat and other animal proteins may produce proinflammatory factors [60]. Chronic inflammation can damage the health of the heart and blood vessels, increasing the risk of cardiovascular disease.

Nonetheless, it is crucial to be mindful of the quantity and quality of carbohydrates and proteins. It is recommended that people choose high-quality sources [61]. Excessive intake of carbohydrates and consumption of low-quality proteins may adversely affect cardiovascular health [62]. This study found that increasing carbohydrate and protein intake can help protect against AF. However, this does not mean higher intake levels will not negatively affect health. Therefore, our research results cannot be generalized to higher intake levels. In future studies, we need to explore the effects of different carbohydrate and protein intake levels on health to better guide people's dietary choices. While our research has discussed potential mechanisms, it is important to acknowledge that these findings are speculative and lack robust evidence. Therefore, caution is necessary when interpreting and generalizing these results. Essential mechanistic studies are needed to investigate the effects of macronutrient intake, quantity, and quality on AF development and progression. Advancing our knowledge in this field will enable us to provide more precise dietary recommendations, empowering individuals to make informed choices for preventing and managing atrial fibrillation.

Our study acknowledges several limitations that merit attention. Firstly, the exploration into the relationship between relative fat intake and AF failed to produce significant findings, a constraint likely stemming from the insufficient number of instrumental variables pertaining to relative fat intake. This limitation restricted the depth of our analyses. Secondly, the absence of high-quality GWAS studies for specific subgroups of relative carbohydrate and protein intake prevented us from performing detailed subgroup analyses. Such analyses could have provided more nuanced insights into the differential effects of various macronutrients on AF risk. Thirdly, the study's focus on individuals of European ancestry limits its generalizability. The findings might not extend to other populations with different genetic backgrounds, lifestyles, and dietary habits, underscoring the need for further research in diverse cohorts to validate and broaden our understanding of the relationship between macronutrient intake and AF risk. Additionally, we recognize the need for robust evidence to support the biological plausibility and validate the speculative links between macronutrient intake and AF, which our study proposes. Future research is essential to explore these potential mechanisms more thoroughly and to address the gaps identified in our study, contributing to a more comprehensive understanding of AF pathogenesis.

## Conclusion

Using MR analysis, we identified a causal relationship between higher predicted relative carbohydrate and protein intake based on genetics and a reduced risk of atrial fibrillation. Higher relative dietary macronutrient intakes were associated with a lower risk of atrial fibrillation. However, further research is needed to confirm these findings and elucidate their underlying mechanisms.

## Data Availability

The summary statistics for relative macronutrient intake and AF are available at the Social Science Genetic Association Consortium (SSGAC; https://www.thessgac.org/data) and IEU open gwas project, respectively.

## Footnotes

1 https://www.acc.org/latest-in-cardiology/articles/2020/03/18/15/42/could-eating-more-protein-reduce-risk-of-afib-in-women-acc-2020

2 https://www.acc.org/about-acc/press-releases/2020/03/19/09/47/eating-more-protein-could-help-ward-off-atrial-fibrillation-in-women#.XqvrZWik070.twitter
